# A Systematic Review of the Interventions for Management of Pain in Patients After Spinal Cord Injury

**DOI:** 10.7759/cureus.42657

**Published:** 2023-07-29

**Authors:** Ioannis Koukoulithras, Abdulaziz Alkhazi, Athanasios Gkampenis, Alexandra Stamouli, Minas Plexousakis, Gianna Drousia, Eleana Xanthi, Charis Roussos, Spyridon Kolokotsios

**Affiliations:** 1 Department of Neurosurgery, University Hospital of Ioannina, Ioannina, GRC; 2 Faculty of Medicine, University of Ioannina, Ioannina, GRC; 3 Department of Neurosurgery, Mater Dei Hospital, Msida, MLT; 4 Faculty of Medicine, University of Malta, Imsida, MLT; 5 Department of Physical Therapy, University Hospital, University of West Attica, Athens, GRC; 6 Faculty of Medicine, National and Kapodistrian University of Athens, Athens, GRC; 7 Department of Physical Medicine and Rehabilitation, Mitera Hospital, Athens, GRC

**Keywords:** pharmaceutical medicines, minimal invasive approach, physiotherapy intervention, antidepressants, anticonvulsants, a systematic review, nociceptive pain, neuropathic pain, chronic pain, spinal cord injury

## Abstract

Chronic pain is a very common problem in patients with spinal cord injury (SCI) as it affects 80% of these patients, which negatively affects their quality of life. Despite many advantages that exist in the management of any type of pain (neuropathic, nociceptive, mixed) in these patients, there is no cure, and the analgesic effect of some treatments is inadequate.

This study aims to conduct an evidence-based systematic review regarding the various interventions used for the management of pain after SCI. The PubMed, Physiotherapy Evidence Database (PEDro), and Cochrane Library databases were searched from 1969 to 2023. The risk of bias was assessed using the PEDro scoring system.

A total of 57 studies met the inclusion criteria and were included in this systematic review. Among the different interventions at present, 18 studies examined the role of oral medications, 11 studies examined the role of minimally invasive methods (injection and infusion), 16 studies investigated physiotherapy and alternative treatments, and 12 studies examined the role of repetitive transcranial magnetic stimulation (rTMS), transcranial direct current stimulation (tDCS), and cranial electrotherapy stimulation (CES) in the management of pain in patients after SCI.

Gabapentin and pregabalin are very effective in managing chronic neuropathic pain after SCI, and pregabalin also seems to reduce anxiety and sleep disturbances in the patients. It is noteworthy that lamotrigine, valproate, and carbamazepine do not have an analgesic effect, but mirogabalin is a novel and promising drug. Antidepressants (selective serotonin reuptake inhibitors and serotonin and noradrenaline reuptake inhibitors) did not reduce the pain of the patients, although some studies showed an efficacy of amitriptyline especially in depressed patients and tramadol should be considered short-term with caution. Also, tDCS and rTMS reduced pain. Moreover, botulinum toxin type A, lidocaine, ketamine, and intrathecal baclofen significantly reduced pain intensity, although the sample of the studies was small. Physiotherapy and alternative treatments seem to relieve pain, and transcutaneous electrical nerve stimulation had the greatest reduction of pain intensity.

In conclusion, several pharmaceutical and non-pharmaceutical methods exist, which can reduce pain in patients after SCI. The type of intervention can be considered by the physician depending on the patients' preference, age, medical history, type of pain, and associated symptoms. However, more studies with greater samples and with better methodological quality should be conducted.

## Introduction and background

Pain is a very common problem for patients with spinal cord injuries (SCIs), with up to 80% of these patients experiencing chronic pain that negatively affects their quality of life. Many factors may contribute to chronic pain, such as damage to nerves, increased nerve impulses, molecular changes in spinal cord receptors, functional changes in supraspinal and cortex structures, and inflammation that occurs in the spinal cord [1,2]. Patients with SCI often report different types of pain. The pain may be a nociceptive type or a neuropathic type or both. Neuropathic pain is a type of pain that is caused by damage to the nervous system, while nociceptive pain is a type of pain that is caused by damage to the non-neural tissue [1]. Neuropathic pain seems to be the most common type of pain in SCI patients and seems persistent despite the existing treatment options. Also, musculoskeletal pain, which is the most common source of nociceptive pain, especially in patients with incomplete SCI, can be treated with non-steroidal anti-inflammatory drugs (NSAIDs) [3,4].

Treating patients with SCI can be challenging for the doctor, as it can be accompanied by several severe impairments, including paralysis, sensory loss, neurogenic bowel, bladder function, and chronic pain [4]. Regardless of the type of pain, chronic pain can significantly impact functioning, mood, and life satisfaction [3].

Many studies and systematic reviews have revealed that antidepressants (e.g., amitriptyline) and anticonvulsants (e.g. pregabalin) are the first-line treatments for neuropathic pain. Many other invasive or non-invasive methods have been proposed, although most have not been appropriately researched [3,4]. This systematic review aims to examine all the available management methods, both invasive and non-invasive, used to treat SCI-related pain and combine their effectiveness.

Material and methods

This systematic review was conducted in compliance with the Preferred Reporting Items for Systematic Reviews and Meta-Analysis (PRISMA) guidelines. This study is an update and extension of Boldt et al. [5] and Mehta et al., [6], who investigated the pharmacological management of pain after SCI. This systematic review aims to investigate all the methods used to manage chronic pain in patients after SCI.

Search Strategy

The literature search was conducted to allocate all studies through PubMed, Physiotherapy Evidence Database (PEDro), and Cochrane Library databases. Studies between 1969 and 2023 were screened. Only clinical trials and randomized controlled trials were included in the study according to PRISMA guidelines. The search strategy was based on the following MeSH (Medical Subject Headings) terms: ("Spinal Cord Injuries"[Mesh]) AND "Pain"[Mesh]). Firstly, we screened the title and the abstract of the studies and then assessed the full text of the potentially eligible articles. Studies were included in this systematic review if they were written in English or Greek, had more than four patients who had an SCI, and if any intervention was used (pharmaceutical, minimally invasive, surgical, physical therapy, etc.) for the management of pain.

Exclusion Criteria

The exclusion criteria were established from the beginning of this systematic review. Other articles except randomized controlled trials or clinical trials were not included in this study. Also, articles written in different languages except English and Greek, studies with less than four patients, and articles with no interventions were not included, as well as articles that did not have full text available.

Methodological Quality

The methodological quality of the included studies was assessed by all the authors independently using the PEDro scoring system [7]. This tool assesses 11 criteria. The maximum total score is 10. To evaluate the quality of the studies, the total PEDro score was categorized as <<low>> for articles with scores less than 4, <<moderate>> for articles with scores between 4 and 6, and <<high>> with scores between 7 and 10.

Data Extraction

The data were extracted from the articles using an electronic sheet, which included the authors' name and date, experimental group, control group, study design, duration of intervention, number of participants, type of pain (neuropathic, nociceptive, mixed), type of injury, and the outcomes.

## Review

Results

Literature Review Results

**Figure 1:**
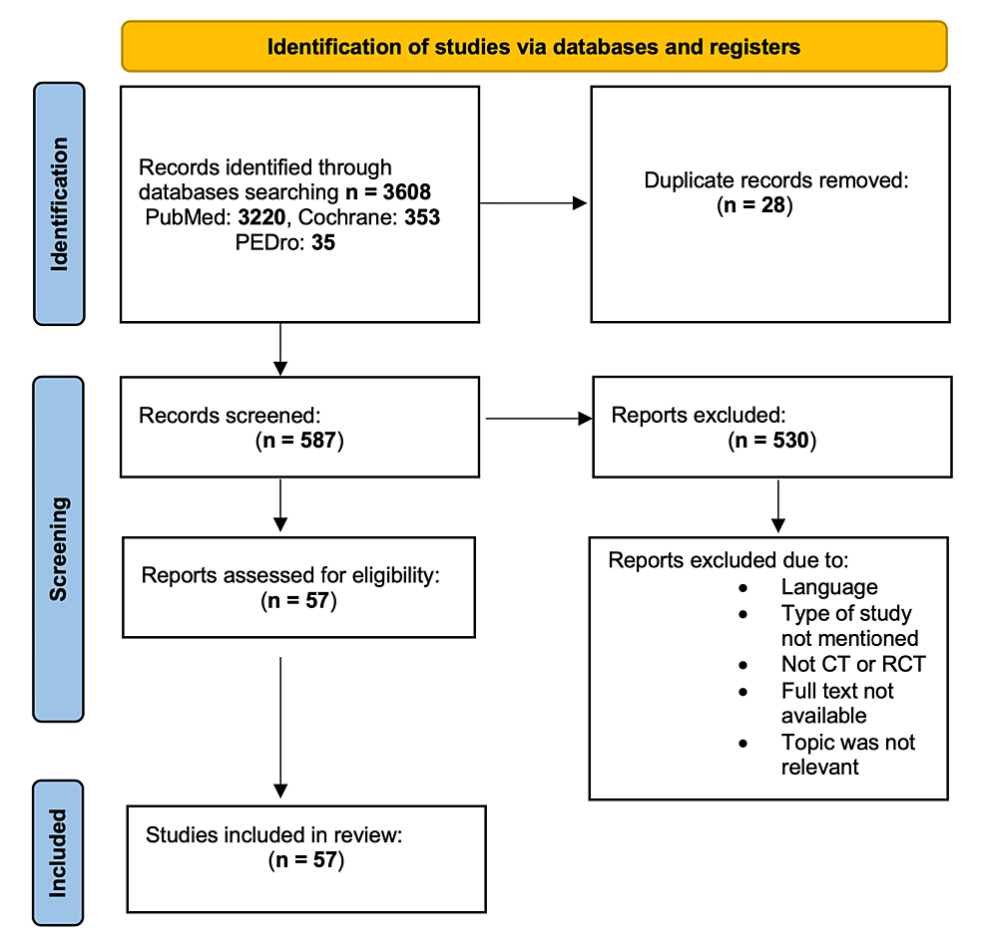
Preferred Reporting Items for Systematic Reviews and Meta-Analyses (PRISMA) flow diagram Abbreviations: CT: clinical trial, RCT: randomized controlled trial

The study selection and systematic review processes are present in the flow diagram (Figure 1). From the initial database search, 3,608 articles were collected (PubMed: 3220, Cochrane: 353, Pedro: 35). Articles were reduced to 3,580 after removing duplications (28 total articles). Based on the inclusion and exclusion criteria mentioned previously and the design of this systematic review, a total of 587 records were screened. Only 57 studies (clinical trials and randomized controlled trials) were eligible to be included in this systematic review, with a total of 2,234 patients. Overall, 530 studies were excluded because they were written in different languages, the authors did not mention the type of the study, the type of the study was not eligible, full text was not available, and the topic of the articles was not relevant. The included articles are listed in Table 1.

**Table 1:** Characteristics of the included studies Abbreviations: ASIA, American Spinal Injury Association; BFA, battlefield acupuncture; CBT - PM, cognitive behavioral therapy pain management programs; CES, cranial electrotherapy stimulation; CHEPs, contact heat-evoked potentials; CMI, clinical meditation and imagery; CNS, Central Nervous System; CONT, control; GI, guided imagery; HMD, head-mounted; i.t. baclofen, intrathecal baclofen; ITB, intrathecal baclofen; LF-TENS, low-frequency transcutaneous electrical nerve stimulation; MI, mental imagery; MS, multiple sclerosis; MT, massage therapy; NP, neuropathic pain; NRS, Numerical Rating Scale; OMT, osteopathic manipulative treatment; PGE2, prostaglandin E2; QOL, quality of life; RCT, randomized controlled trial; rTMS, repetitive transcranial magnetic stimulation; SCI, spinal cord injury; SRP, spasm-related pain; tDCS, transcranial direct current stimulation; TENS, transcutaneous electrical nerve stimulation; VI, visual illusion; VR, virtual reality; XR, extended release

Study	Experimental group	Control group	Study design	Duration of intervention	Number of participants	Type of pain (neuropathic, nociceptive, mixed)	Type of injury	Outcomes
Funda Levendoglu et al., 2004 [8]	Gabapentin	Placebo	Prospective, randomized, double-blind, placebo-controlled, crossover clinical trial	18 weeks	20	NP	Paraplegic patients with complete traumatic SCI at the thoracic and lumbar level	Gabapentin reduced the intensity as well as the frequency of pain, relieved all NP descriptors except the itchy, sensitive, dull, and cold types, and improved the QOL
Ushida et al., 2023 [9]	Mirogabalin	Placebo	Randomized, double-blind, placebo-controlled	14 weeks	120	Central NP	Traumatic SCI	Mirogabalin elicited clinically relevant decreases in pain
Cardenas et al., 2013 [10]	Pregabalin	Placebo	RCT	17 weeks	220	NP	SCI	Pregabalin is effective and well tolerated in patients with NP due to SCI
Salinas et al., 2012 [11]	Carbamazepine	Placebo	Randomized, double-blind, placebo-controlled clinical trial	24 weeks	46	NP	SCI	Early intervention with carbamazepine decreased NP incidence at the 1-month follow-up but not at the 3- and 6-month follow-ups in patients with acquired SCI
Siddall et al., 2006 [12]	Pregabalin	Placebo	Placebo-controlled trial	12 weeks	90	Central NP	SCI	Pregabalin 150 to 600 mg/day was effective in relieving central NP and improving sleep, anxiety, and overall patient status in patients with SCI.
Ahn et al., 1976 [13]	Gabapentin		Clinical trial	8 weeks	31	NP	SCI or cauda equina syndrome	Gabapentin may be effective in decreasing NP refractory to conventional analgesics in some patients with SCI whose duration of symptoms is less than 6 months, although those with a duration of symptoms longer than 6 months showed a significant decrease as well
Tai et al., 2002 [14]	Gabapentin	Placebo	Prospective, randomized, double-blind, crossover, and placebo-controlled clinical trial	10-week	7	NP	SCI	Gabapentin has some beneficial effects on certain types of NP. There was a significant decrease in "unpleasant feeling" and a trend toward a reduction of both the "pain intensity" and "burning sensation" at the fourth week of gabapentin treatment compared with those on the placebo
Finnerup et al., 2002 [15]	Lamotrigine	Placebo	Randomized double-blind, crossover trial	9 weeks	30	NP	SCI	While this trial showed no significant effect on spontaneous and evoked pain in complete and incomplete SCI, lamotrigine reduced spontaneous pain in patients with incomplete SCI and evoked pain in the area of spontaneous pain
Drewes et al., 1994 [16]	Valproate	Placebo	Double-blind cross-over study	8 weeks	20	NP	SCI	No significant analgesic effects of valproate could be demonstrated although serum concentration and dose reached a high level.
Rintala et al., 2007 [17]	Gabapentin or amitriptyline	Diphenhydramine	RCT double-blind cross-over	8 weeks	38	NP	SCI	Amitriptyline seemed to be effective in relieving chronic pain after SCI, especially in patients with depressive symptomatology
Amr, 2010 [18]	Multi-day low-dose ketamine infusion as adjuvant to oral gabapentin	Placebo	Randomized, controlled, double-blind trial	4 weeks	40	NP	SCI	Multi-day low-dose ketamine infusion as adjuvant to gabapentin in post-SCI-related chronic pain is safe and efficacious in reducing pain, but the effect compared to placebo ceased 2 weeks after infusion termination
Agarwal and Joshi, 2017 [19]	Amitriptyline	Lamotrigine	Randomized longitudinal comparative study	3 weeks	140	NP	Traumatic SCI	The present study demonstrated the effectiveness of both amitriptyline and lamotrigine in SCI-induced NP; no difference in efficacy was noted between the two drugs
Richards et al., 2015 [20]	Venlafaxine XR	Placebo	Randomized, controlled trial	12 weeks	123	NP	SCI of any level;	Venlafaxine XR for treating central NP is likely to be limited
Vranken et al., 2011 [21]	Duloxetine	Placebo	Randomized, double-blind, placebo-controlled clinical trial	8 weeks	48	NP	SCI	No significant effect on pain intensity, duloxetine revealed a biological effect.
Cardenas et al., 2002 [22]	Amitriptyline	Placebo	Double-blind, randomized, trial	6 weeks	84	Chronic pain	SCI	No significant differences were found between the groups in pain intensity or pain-related disability posttreatment in either intent-to-treat analyses or analyses of study completers
Chiou-Tan et al., 1996 [23]	Mexiletine	Placebo	Prospective, randomized, double-blind, crossover design trial.		15	NP	SCI	In conclusion, in this trial, mexiletine did not appear to decrease SCI-related dysesthetic pain
Zarepour al., 2020 [24]	Bumetanide	Placebo	RCT	19 weeks	9	NP	Traumatic SCI	Bumetanide treatment significantly reduced average pain intensity according to the NRS and the short form of the McGill Pain Questionnaire scores
Rintala et al., 2010 [25]	Dronabinol (cannabinoid)	Diphenhydramine	RCT double-blind cross-over	30 weeks	7	NP	ASIA A, B, C, D	Dronabinol was no more effective than diphenhydramine
Norrbrink and Lundeberg, 2009 [26]	Tramadol	Placebo	Randomized, double-blind, placebo-controlled trial	4 weeks	36	NP	SCI	Tramadol might be tried for NP after SCI after the use of gabapentin/pregabalin, and tricyclic antidepressants have been found to be insufficient. Titration should be slow and individual to minimize the risk of adverse events.
Kvarnström et al., 2004 [27]	Intravenous ketamine and lidocaine	Placebo	Randomized, double-blind, three-period, three-treatment, cross-over design		10	NP	Traumatic SCI	Ketamine but not lidocaine showed a significant analgesic effect in patients with NP after SCI
Chun et al., 2019 [28]	Injection of botulinum toxin A	Injection of normal saline (placebo)	Randomized, double-blind, placebo-controlled, cross-over study	12 weeks	4	NP	Chronic traumatic SCI	Not a statistical significance, but a higher proportion of participants reported a marked change in average pain intensity from baseline to 8 and 12 weeks post-botulinum toxin type A vs. post-placebo
Kumru et al., 2018 [29]	Single ITB bolus	Placebo	Double-blind, placebo-controlled study		11	NP	Complete or incomplete SCI of traumatic or non-traumatic etiology at the cervical or thoracic level	An ITB bolus exerted a significant analgesic effect on all subtypes of NP in SCI patients
Li et al., 2017 [30]	Botulinum toxin A	Placebo	RCT	8 weeks	41	NP	SCI with NP (A - D level at ASIA classification)	Botulinum toxin type A might decrease intractable NP in patients with SCI
Han et al., 2016 [31]	Botulinum toxin A	Placebo	Randomized, double-blind, placebo-controlled design	8 weeks	49	NP	SCI of any level	Botulinum toxin type A may reduce intractable chronic NP in patients with SCI
Finnerup NB et al., 2006 [32]	Lidocaine infusion	Placebo	Randomized, double-blind, placebo-controlled, crossover trial	2 weeks	24	NP	SCI	Lidocaine reduced NP at and below the level of injury irrespective of the presence or absence of evoked pain.
Siddall et al., 2000 [33]	Intrathecal infusion of morphine or clonidine, alone or combined	Placebo	Double-blind, randomized, controlled trial		15	NP	SCI	In conclusion, intrathecal administration of morphine and clonidine appears to provide good pain relief for a proportion of patients with NP after SCI who are unresponsive to other interventions
Attal et al., 2000 [34]	Intravenous lidocaine	Placebo	Double-blind, placebo-controlled, psychophysical study (CT)		16	Central pain	SCI	Systemic lidocaine can induce a significant and selective reduction of several components of pain caused by CNS injuries
Loubser and Akman, 1996 [35]	ITB		Clinical trial	12 weeks	16	Chronic pain was delineated into neurogenic and musculoskeletal components	SCI	This study suggests that ITB reduces chronic musculoskeletal pain associated with spasticity but does not decrease chronic neurogenic SCI pain.
Herman et al., 1992 [36]	ITB	Placebo	Double-blind, randomized, controlled trials		9	Chronic, dysesthetic, and spasm-related pain	Spinal spasticity, i.e., MS, SCI, transverse myelitis	ITB caused a marked reduction of segmental reflexes before the suppression of intersegmental reflexes, significantly suppressed dysesthetic pain and SRP with temporal dissociation, and did not influence pinch-induced and musculoskeletal (low back) pain
Zhao et al., 2020 [37]	rTMS	Sham	RCT	3 weeks	48	NP	37 patients complete SCI, 11 patients incomplete SCI	rTMS alleviated NP statistically significantly in all pain assessment scales in patients with acute SCI. For long-lasting clinical effects, it should be used in combination with pharmacological approaches
Yeh et al., 2021 [38]	Real tDCS + upper-body exercises	Sham tDCS + upper-body exercises	Double-blind RCT	12 sessions of real or sham tDCS and a 4-week follow-up	12	NP	Chronic SCI	The effect of tDCS with exercise was not significantly superior to exercise alone immediately after the intervention. The beneficial effects appeared after a period of time (follow-up)
Kang et al., 2009 [39]	rTMS	Sham rTMS	RCT	12 weeks	11	NP	ASIA A, B, C, D	There was no therapeutic efficacy of real rTMS than sham when rTMS was applied in the hand motor cortical area of the brain
Defrin et al., 2007 [40]	rTMS	Sham rTMS	RCT	4,5 weeks	11	NP	SCI complete or incomplete	Real rTMS and sham seemed to reduce chronic pain statistically significantly (p<0.05) after 10 sessions
Sun et al., 2019 [41]	rTMS pulses	Sham	Double-blind, sham-controlled, clinical trial	6 weeks	21	NP	Complete or incomplete SCI	The real rTMS, compared with the sham, showed more pain relief from two weeks to six weeks. The pain intensity was not remarkably changed at week 1.
Nardone et al., 2017 [42]	Active rTMS	Sham rTMS	RCTl	2 weeks	12	NP	Cervical or thoracic SCI	Active rTMS had a statistically significant reduction in pain symptoms compared to sham rTMS
Yılmaz et al., 2014 [43]	rTMS	Sham rTMS	Randomized, double-blind, clinical trial	Every day for 10 days	17	NP	Chronic SCI	The analgesic effect of rTMS on intractable NP in SCI was not superior to sham. However, middle-term (over 6 weeks) pain relief by rTMS is encouraging
Jetté et al., 2013 [44]	rTMS	Sham	Double-blind, cross-over randomized study	2 weeks	16	NP	A total of 16 patients with complete or incomplete motor SCI	RTMS applied over the hand or leg motor cortex decreased NP regardless of any change in cortical excitability, suggesting that the analgesic effect is not associated with local changes at the motor cortex level itself
Kumru et al., 2013 [45]	tDCS +VI	14 healthy	Clinical trial	2 weeks	18	NP	SCI	Two weeks of tDCS + VI induced significant changes in CHEPs, evoked pain and heat pain threshold in SCI patients with NP
Soler et al., 2010 [46]	tDCS	Sham	Randomized, double-blind, sham-controlled clinical trial	12 weeks	39	NP	SCI	Results demonstrate that tDCS and VI can be effective in the management of NP following SCI, with minimal side effects and with good tolerability.
Tan et al., 2006 [47]	CES	Placebo	placebo-controlled trial	5 weeks	38	Chronic pain	SCI	CES can effectively treat chronic pain in persons with SCI
Yoon et al., 2014 [48]	tDCS	Sham tDCS	RCT	10 days (20 minutes, 2 mA, twice a day)	16	NP	ASIA A, B	Anodal stimulation of the motor cortex using tDCS can modulate emotional and cognitive components of pain and normalize excessive attention to pain and pain-related information
Zanca et al., 2022 [49]	Clinical meditation and imagery	Education on topics related to health and function after SCI	RCT	8 weeks	24	Both neuropathic and nociceptive pain types	Chronic traumatic or non-traumatic SCI (duration of injury > 1 year)	Both groups showed relatively low levels of pain interference at baseline, but pain interference decreased to a greater extent in the CONT group than in CMI
Austin et al., 2021 [50]	3D HDM VR	2D screen applications using the same virtual environment	Randomized cross-over pilot trial		16	NP	Complete or incomplete SCI of longer than 12 months duration, lesion at C6 level or below	3D HMD VR may provide NP relief for people with SCI
Mulroy et al., 2011 [51]	Strengthening and optimal movements	Placebo	Randomized controlled clinical trial	12 weeks	80	Painful shoulders	SCI	This home-based intervention was effective in reducing long-standing shoulder pain in people with SCI. The reduction in pain was associated with improvements in muscle strength and health-related and overall QOL
Hicks et al., 2003 [52]	Exercise training	Non-exercising CONT group	RCT	9 months	7		SCI	Long-term twice-weekly exercise training in this population is feasible and results in significant gains in both physical and psychological well-being
Arienti et al., 2011 [53]	OMT	OMT + pregabalin or only pregabaline	RCT	12 weeks	47	NP, nociceptive pain	ASIA A, B, C, D	Patients in the pregabalin group had a 24% improvement, and the osteopathic group had a 16% improvement after 3 weeks. The combination of pregabalin and OMT yielded significantly better pain relief.
Jensen et al., 2009 [54]	Self-hypnosis training	Biofeedback relaxation	RCT	10 sessions	37	NP, non - NP	SCI	Participants who underwent hypnosis reported statistically significant decreases in daily average pain. The analgesia was maintained at the 3-month follow-up.
Bi et al., 2015 [55]	TENS	Sham TENS	RCT	12 weeks	52	Pain (not specific)	SCI	TENS decreased significantly pain after the treatment in patients with SCI
Vitalii and Oleg, 2014 [56]	LF-TENS	Sham TENS	RCT	10 days (30 minutes per session)	25	NP	SCI	LF-TENS may be effective in combination with gabapentin
Kaur et al., 2020 [57]	MI	Sham	RCT	30 minutes, 5 days a week for 4 weeks.	42	NP	Complete and incomplete SCI injury	Significant reductions in total scores of NP in the MI group compared to the CONT group
Burke et al., 2019 [58]	Internet delivery of CBT - PM	Sham	RCT	12 weeks	69	NP	Complete or incomplete SCI	Internet-delivered CBT-PMP reports significant statistical and clinical benefits in pain intensity and interference
Lovas et al., 2016 [59]	MT	GI relaxation	RCT	5 weeks	40	NP	Chronic SCI	Pain scores were reduced significantly over time in both MT and GI groups (p=0.049 and p=0.032)
Allison et al., 2016 [60]	Anti-inflammatory diet	No intervention	Randomized clinical trial	12 weeks	20	NP	SCI of any level or severity	The study demonstrates the efficacy of targeting inflammation as a means of treating NP in SCI, with a potential mechanism relating to the reduction in proinflammatory cytokines and PGE2
Andresen et al., 2016 [61]	Ultramicronized palmitoylethanolamide	Placebo	Randomized, double-blind, placebo-controlled trial	12 weeks	73	NP	Complete and incomplete SCI injury	There was no difference in mean pain intensity between PEA-um and placebo treatment (P = 0.46)
Estores et al., 2017 [62]	Auricular acupuncture, battlefield acupuncture	Delayed acupuncture group	Pilot-controlled clinical trial	8 weeks	24	NP	Complete and incomplete SCI injury	Both groups reported a significant reduction in pain during the trial period, but the BFA group reported more pain reduction than the delayed entry group
Özkul et al., 2015 [63]	Visual illusion application, TENS application	VI application, TENS application	Randomized controlled cross-over trial	5 weeks	24	NP	Traumatic SCI	TENS and VI applications on patients with NP after SCI were found to be helpful. Both applications can be administered as a therapeutic approach.
Celik et al., 2013 [64]	LF-TENS	Placebo	RCT	10 days	33	NP	SCI	This study revealed that in the treatment of NP of SCI patients, LF-TENS may be effective.

A total of 57 studies investigated the management of chronic pain after SCI. Most interventions were pharmacological and minimally invasive (29 studies). Also, the quality of these studies is characterized as “moderate” to “high.” Physiotherapy and alternative treatments were used, as well as transcranial magnetic stimulation (TMS), transcranial direct current stimulation (tDCS), and cranial electrotherapy stimulation (CES). The authors characterized these methods as having “moderate” methodological quality. Table 2 to presents the methodological quality of included studies.

**Table 2:** PEDro scale for the methodological quality of the included studies

Studies	Eligibility criteria	Random allocation	Concealed allocation	Baseline comparability	Blind subjects	Blind therapists	Blind assessors	Adequate follow-up	Intention-to-treat analysis	Between-group comparisons	Point estimates and variability	/10
Burke et al., 2019 [58]	Yes	Yes	No	Yes	No	No	Yes	No	Yes	Yes	Yes	6
Arienti et al., 2011 [53]	Yes	Yes	No	Yes	No	No	No	No	No	Yes	Yes	4
Estores et al., 2017 [62]	Yes	Yes	No	Yes	No	No	No	Yes	No	Yes	Yes	5
Zanca et al., 2022 [49]	Yes	Yes	No	Yes	No	No	Yes	No	No	Yes	Yes	5
Jensen et al., 2009 [54]	Yes	Yes	No	No	No	No	No	Yes	Yes	Yes	Yes	5
Lovas et al., 2016 [59]	Yes	Yes	No	Yes	No	No	No	No	No	Yes	Yes	4
Tan et al., 2006 [47]	Yes	Yes	No	Yes	Yes	Yes	Yes	Yes	No	Yes	Yes	8
Bi et al., 2015 [55]	Yes	Yes	No	Yes	Yes	No	Yes	Yes	No	Yes	Yes	7
Özkul et al., 2015 [63]	Yes	Yes	No	Yes	No	No	No	Yes	No	Yes	Yes	5
Celik et al., 2013 [64]	Yes	Yes	No	Yes	No	No	No	No	No	Yes	Yes	4
Zhao et al., 2020 [37]	Yes	Yes	Yes	Yes	Yes	Yes	Yes	Yes	No	Yes	Yes	9
Sun et al., 2019 [41]	Yes	Yes	No	Yes	Yes	No	Yes	Yes	No	Yes	Yes	7
Nardone et al., 2017 [42]	Yes	Yes	No	Yes	Yes	Yes	Yes	Yes	Yes	Yes	Yes	9
Yılmaz et al., 2014 [43]	Yes	Yes	No	Yes	Yes	No	Yes	Yes	No	Yes	Yes	7
Jetté et al., 2013 [44]	Yes	Yes	No	No	Yes	No	Yes	Yes	No	Yes	Yes	6
Defrin et al., 2007 [40]	Yes	Yes	No	Yes	Yes	No	Yes	Yes	No	Yes	Yes	7
Kang et al., 2009 [39]	Yes	Yes	No	Yes	Yes	No	Yes	Yes	No	Yes	Yes	7
Yeh et al., 2021 [38]	Yes	Yes	No	Yes	Yes	No	Yes	Yes	No	Yes	Yes	7
Yoon et al., 2014 [48]	Yes	No	No	Yes	Yes	No	Yes	Yes	No	Yes	Yes	6
Kumru et al., 2018 [29]	Yes	No	No	Yes	No	No	Yes	Yes	No	Yes	Yes	5
Soler et al., 2010 [46]	Yes	Yes	No	Yes	Yes	No	Yes	Yes	No	Yes	Yes	7
Kvarnström et al., 2004 [27]	Yes	Yes	Yes	Yes	Yes	Yes	No	Yes	No	Yes	Yes	8
Chun et al., 2019 [28]	Yes	Yes	Yes	Yes	Yes	Yes	No	Yes	No	Yes	Yes	8
Kumru et al., 2018 [29]	Yes	Yes	No	Yes	Yes	Yes	Yes	Yes	No	Yes	Yes	8
Li et al., 2017 [30]	Yes	Yes	Yes	Yes	Yes	Yes	Yes	Yes	Yes	Yes	Yes	10
Han et al., 2016 [31]	Yes	Yes	Yes	Yes	Yes	Yes	Yes	Yes	Yes	Yes	Yes	10
Amr, 2010 [18]	Yes	Yes	Yes	Yes	Yes	Yes	No	Yes	No	Yes	Yes	8
Yeh et al., 2005 [38]	Yes	Yes	Yes	Yes	Yes	Yes	No	Yes	No	Yes	Yes	8
Siddall et al., 2000 [33]	Yes	Yes	Yes	Yes	Yes	Yes	Yes	Yes	No	Yes	Yes	9
Attal et al., 2000 [34]	Yes	Yes	Yes	Yes	Yes	Yes	No	Yes	No	Yes	Yes	8
Loubser and Akman, 1996 [35]	Yes	No	No	No	No	No	No	Yes	No	No	Yes	2
Herman et al., 1992 [36]	Yes	Yes	Yes	Yes	Yes	Yes	No	No	No	Yes	Yes	7
Austin et al., 2021 [50]	Yes	Yes	Yes	Yes	No	No	No	Yes	Yes	Yes	Yes	7
Kaur et al., 2020 [57]	Yes	Yes	Yes	Yes	No	No	Yes	Yes	Yes	Yes	Yes	8
Allison et al., 2016 [60]	Yes	Yes	No	Yes	No	No	No	Yes	Yes	Yes	Yes	7
Andresen et al., 2016 [61]	Yes	Yes	Yes	Yes	Yes	Yes	Yes	No	Yes	Yes	Yes	9
Mulroy et al., 2011 [51]	Yes	Yes	Yes	Yes	No	No	Yes	No	Yes	Yes	Yes	7
Hicks et al., 2003 [52]	No	Yes	No	Yes	No	No	No	Yes	No	Yes	Yes	5
Vitalii and Oleg, 2014 [56]	No	Yes	No	Yes	No	No	Yes	No	No	No	Yes	4
Levendoglu et al. 2004 [8]	Yes	Yes	Yes	Yes	Yes	Yes	No	Yes	No	Yes	Yes	8
Ushida et al., 2023 [9]	Yes	Yes	Yes	Yes	Yes	Yes	No	Yes	Yes	Yes	Yes	9
Zarepour et al., 2020 [24]	Yes	No	No	Yes	No	No	No	No	No	No	Yes	2
Agarwal and Joshi, 2017 [19]	Yes	Yes	No	Yes	No	No	No	Yes	No	No	Yes	3
Richards et al., 2014 [20]	Yes	Yes	Yes	Yes	Yes	Yes	Yes	No	No	Yes	Yes	8
Cardenas et al., 2013 [10]	Yes	Yes	Yes	Yes	Yes	Yes	Yes	No	Yes	Yes	Yes	9
Salinas et al., 2012 [11]	Yes	Yes	Yes	Yes	Yes	Yes	No	Yes	No	Yes	Yes	8
Vranken et al., 2011 [21]	Yes	Yes	Yes	Yes	Yes	Yes	No	Yes	Yes	Yes	Yes	9
Norrbrink and Lundeberg, 2009 [26]	Yes	Yes	Yes	Yes	Yes	Yes	Yes	Yes	Yes	Yes	Yes	10
Siddall et al., 2006 [12]	Yes	Yes	Yes	Yes	Yes	Yes	Yes	Yes	Yes	Yes	Yes	10
Ahn et al., 2003 [13]	Yes	Yes	No	Yes	Yes	No	No	Yes	No	Yes	Yes	6
Tai et al., 2002 [14]	Yes	Yes	Yes	Yes	Yes	Yes	No	Yes	No	Yes	Yes	8
Finnerup et al., 2002 [15]	Yes	Yes	Yes	Yes	Yes	Yes	Yes	Yes	Yes	Yes	Yes	10
Cardenas et al., 2002 [22]	Yes	Yes	Yes	Yes	Yes	Yes	Yes	Yes	Yes	Yes	Yes	10
Chiou-Tan et al., 1996 [23]	Yes	Yes	Yes	Yes	Yes	Yes	No	Yes	No	Yes	Yes	8
Drewes et al., 1994 [16]	Yes	Yes	Yes	Yes	Yes	Yes	No	Yes	No	Yes	Yes	8
Rintala et al., 2010 [25]	Yes	Yes	Yes	Yes	Yes	Yes	No	Yes	No	Yes	Yes	8
Rintala et al., 2007 [17]	Yes	Yes	Yes	Yes	Yes	Yes	Yes	Yes	No	Yes	Yes	9

Many oral medicines are used for the management of chronic pain. Gabapentin, mirogabalin, pregabalin, carbamazepine, lamotrigine, and valproate were the anticonvulsants found in the included studies [8-17]. One study researched the low-dose ketamine infusion as an adjuvant to oral gabapentin [18]. Moreover, the antidepressants studied were amitriptyline, venlafaxine, duloxetine, and mexiletine [17,19-23]. Also, one study investigated the role of bumetanide, which is a diuretic drug [24], and only one randomized controlled trial was found to investigate the role of dronabinol, which is a cannabinoid [25]. Furthermore, tramadol (opioid) was examined in one study [26].

The minimally invasive methods include intravenous ketamine and lidocaine injections, botulinum toxin A injection, intrathecal baclofen injection/infusion, lidocaine infusion, and intrathecal infusion of clonidine and morphine [27-36].

TMS and tDCS were studied in 12 articles [37,38,47,48,39-46].

We found a total of 16 studies that assessed physiotherapy and alternative methods for the management of pain. Clinical meditation and imagery intervention, 3D head-mounted virtual reality, mental imagery, exercise, cognitive behavioral therapy (CBT), massage, anti-inflammatory diet, ultra micronized palmitoylethanolamide, auricular acupuncture, transcutaneous electrical nerve stimulation (TENS), orthopedic manipulative therapy (OMT), and self-hypnosis were among these included studies [49,50,59-64,51-58].

Discussion

Anticonvulsants

Anticonvulsants have been used for decades as a first-line treatment for neuropathic pain as many studies have revealed their effectiveness in many patients. Gabapentin seems to be more effective than other anticonvulsants such as pregabalin, mirogabalin, lamotrigine, carbamazepine, and valproate.

Levendoglu et al. [8] have shown that gabapentin is the first-line medication for treating neuropathic pain in patients that have SCIs. Also, Ahn et al. [13] affirmed the effectiveness of gabapentin in reducing neuropathic pain after SCI especially if the duration of the injury was less than six months. Moreover, Tai et al. [14] also investigated the efficacy of gabapentin in reducing neuropathic pain after SCI. They concluded that gabapentin can reduce some types of neuropathic pain in patients with SCI, although it can cause dizziness and drowsiness in some patients, especially in the elderly, and thus it should be administrated with caution.

Pregabalin was the second most common medication used in the studies for treating neuropathic pain after SCI. Cardenas et al. [10] conducted a randomized controlled trial with various doses of pregabalin for treating neuropathic pain after SCI. They found a statistically significant change when compared to a placebo in improving pain throughout the trial period. They, therefore, concluded that pregabalin was well tolerated, and the medication was also effective in treating neuropathic pain due to SCIs. Also, Siddall et al. [12] in their study found that pregabalin reduced neuropathic pain as well as seemed to improve anxiety and sleep in patients.

On the other hand, in most of the studies, lamotrigine, valproate, and carbamazepine were ineffective in managing neuropathic pain in SCI patients. Finnerup et al. [15] investigated the effectiveness of lamotrigine. They conducted a randomized, double-blind controlled trial and concluded that lamotrigine did not reduce both evoked and spontaneous pain in patients with both complete and incomplete SCI, as opposed to the usual belief that lamotrigine use is associated with reduced pain in both patients with incomplete and complete SCI. Also, Salinas et al. [11], who conducted a randomized, placebo-controlled, double-blind trial, concluded that carbamazepine only effectively reduced neuropathic pain at one month. However, longer-term pain control at three and six months was not demonstrated in patients with neuropathic pain [29].

Drewes et al. [16], who investigated the use of valproate in a double-blind, cross-over study in treating severe chronic central pain, found no analgesic effect of the medication and advised no further studies to explore this.

It is well mentioned that mirogabalin is a novel drug researched by Ushida et al. [9], who conducted a randomized, double-blind, controlled phase 3 trial to determine its safety and efficacy in treating chronic neuropathic pain in patients with traumatic SCI. They concluded that the administration of mirogabalin led to a statistically significant change in the pain score from baseline in patients with SCI and therefore concluded that mirogabalin is a promising drug for treating chronic neuropathic pain following SCI.

Antidepressants

Antidepressants are another category of drugs used daily for managing chronic neuropathic pain and they can be combined in many patients with anticonvulsants. More specifically, serotonin and norepinephrine reuptake inhibitors such as duloxetine and venlafaxine did not show a significant effect in reducing neuropathic pain after SCI, as shown in the studies by Richards et al. [20] and Vranken et al. [21].

The effectiveness of tricyclic antidepressants such as amitriptyline seems to be controversial. The study of Cardenas et al. [22], who investigated the efficacy of amitriptyline, showed that it did not improve pain in six weeks of treatment. On the other hand, Rintala et al. [17] in a randomized, controlled, double-blind, triple cross-over trial compared the effectiveness of gabapentin and amitriptyline and concluded that amitriptyline was as effective as gabapentin. However, in patients with depressive symptoms, amitriptyline seemed more effective than gabapentin in reducing neuropathic pain after eight weeks of therapy. Moreover, Agarwal and Joshi [19], who conducted a randomized longitudinal study to compare the efficacy of lamotrigine and amitriptyline in patients with neuropathic pain following SCI, found a significant difference between the baseline average pain score and the value at follow-up in both the lamotrigine and the amitriptyline groups. Therefore, they concluded that both medications can be used to treat neuropathic pain following SCI.

Tramadol

Tramadol, which is a weak opioid, can be used with caution for a short term for the management of chronic neuropathic pain. Norrbrink et al. [26] conducted a study on the safety and effectiveness of tramadol in relieving neuropathic pain in patients with SCI. They conducted a randomized, placebo-controlled, double-blind trial and concluded that tramadol use was associated with a reduction in pain intensity after four weeks of therapy. However, patients had significant side effects. They, therefore, advised that tramadol can be considered after using tricyclic antidepressants and either pregabalin or gabapentin.

Bumetanide

Zarepour et al. [24] investigated the analgesic properties of bumetanide as an adjunct in managing neuropathic pain in patients with SCI. They conducted an open-label single-arm pilot trial of bumetanide, which was added to treat patients with SCI for 19 weeks. They concluded that the data reiterated the analgesic effect of bumetanide through disinhibiting the GABAergic pathway by upregulating the KCC2 protein.

Mexiletine

Chiou-Tan et al. [23] examined the effect of mexiletine in treating spinal cord dysesthetic pain. They performed a randomized, placebo-controlled, double-blind crossover trial. They found that there was no significant effect on pain in patients with SCI and concluded that mexiletine does not help in pain reduction in these patients.

Dronabinol

Last but not least, only one study investigated the role of dronabinol, which is a cannabinoid in the treatment of chronic neuropathic pain after SCI. Rintala et al. [25] concluded that dronabinol did not outperform diphenhydramine in relieving chronic pain.

tDCS

tDCS seems to be a very promising and safe method for managing chronic neuropathic pain after SCI. In this method, one anode and one cathode electrode are applied to the scalp, and a low sub-threshold electric current is applied to neuromodulate the targeted brain area. According to four of the five included studies, tDCS either alone or in combined visual illusion leads to a significant improvement in neuropathic pain after SCI [38,43-46]. Kumru et al. [45] assessed the pain perception threshold and found that there were significant changes in the evoked potentials after two weeks of tDCS and visual illusion. Soler et al. [46] after assessing different combinations of tDCS, sham tDCS, visual illusion, and control illusion concluded that tDCS combined with visual illusion resulted in the most significant reduction of neuropathic pain intensity. tDCS seems to induce metabolic changes, increasing metabolism in the medulla and subgenual anterior cingulate cortex as well as reducing metabolic activity in the left dorsolateral prefrontal cortex, as shown in a PET study by Yoon et al. [48]. Nevertheless, a study by Yeh et al. [38] showed no superiority of tDCS combined with exercise regarding pain relief.

TMS

TMS aims to interfere with brain circuits generating electricity through the applied magnetic field. rTMS is a kind of TMS that uses repetitive pulses to generate repetitive electric currents in the targeted brain region. In some studies, rTMS seems to significantly reduce the neuropathic pain in SCI patients, as shown in the studies by Nardone et al. [42] and Jetté et al. [44]. Jetté et al. [44] found that corticospinal excitability was increased after stimulation of the hand area, but neuropathic pain was reduced after stimulation over either leg or hand motor cortex. Defrin et al. [40] found that rTMS and sham TMS both significantly and similarly reduced pain intensity. However, only rTMS increased the heat-pain threshold and showed an effect on the follow-up period. Also, two studies included in this systematic review showed intermediate-term pain reduction and no difference between rTMS and placebo long-termly [39,41]. Lastly, a study by Kang et al. [39] regarding rTMS over hand motor cortical area showed no difference in pain relief between rTMS and placebo group, but their measurements were held only one week after rTMS and intermediate and long-term results were not assessed.

Minimally Invasive Methods (Botulinum Toxin Type A, Intravenous Lidocaine, Ketamine, Baclofen, Morphine, and Clonidine)

Moreover, in our systematic review, three studies examined the role of botulinum toxin type A in chronic pain after SCI. Two studies, one performed by Li et al. [30] and the other by Han et al. [22], showed a statistically significant reduction of pain in patients who received the toxin than placebo. In both studies, the patients in the intervention group received subcutaneous 200 units of botulinum toxin A in 4 mL saline solution at the painful area. Li et al. [30] in their study used a sample of 41 patients (21 intervention group, 20 placebo group), Han et al. [31] used a sample of 40 patients (20 intervention group, 20 placebo group) and both studies performed a follow-up at 4 and 8 weeks after the injection. Both studies yielded statistically significant results in neuropathic pain reduction at 4 and 8 weeks of follow-up, demonstrating that botulinum toxin might be a viable treatment for patients with SCI-related chronic pain. The third study was performed by Chun et al. [28] but due to the small sample size (n = 4), the results weren't statistically significant.

Intravenous lidocaine was used in three studies. Finnerup et al. [32] performed a randomized double-blind crossover trial comparing lidocaine with a placebo, and a total of 24 patients with SCI-related pain were examined. Each patient in the intervention group received 5 mg/kg of lidocaine over 30 min. The results were statistically significant, and lidocaine relieved both at-level and below-level neuropathic pain. Attal et al. [34] also studied the effects of lidocaine on patients with SCI pain and concluded that lidocaine is superior to placebo, although the study sample was only six patients therefore the study is considered as low statistical power. Kvarnström et al. [27] studied the effects of ketamine and lidocaine in a randomized, double-blind, cross-over study. Lidocaine did not show an analgesic effect in patients with neuropathic pain, although a small sample size was used (n = 4).

Kvarnström et al. [27] also studied the effects of ketamine on patients with SCI pain. Specifically, 4 mg/kg was given, and the results showed that ketamine reduced neuropathic pain. Amr [18] used ketamine infusion as a complementary therapy to oral gabapentin. Forty patients were examined and the results showed that complementary ketamine infusion significantly reduces neuropathic pain in patients with SCI pain compared to the placebo group but only for two weeks.

The analgesic effects of intrathecal baclofen were examined in three studies. Kumru et al. [29] conducted a randomized, double-blind study with a sample of 11 patients with SCI pain (n = 11). In the intervention group, 50 μg of intrathecal baclofen was used, and 1 mL of physiologic sodium chloride was used in the placebo group. The results showed that a single intrathecal baclofen bolus significantly improved all subtypes of neuropathic pain, but the sample size was small, and the effects were only studied for 24 hours. Loubser and Akman [35] and Herman et al. [36] also studied the effects of intrathecal baclofen on patients with SCI pain, but their results were not statistically significant. Herman et al. [36] used a sample of two patients in their study (n = 2), and Loubser and Akman [35] had a selection bias, lack of a placebo group, and poor pain measurement methods.

Morphine is a potent opioid and clonidine, which is an antihypertensive drug used to treat neuropathic pain. Siddall et al. [33] studied the effects of these two medications on patients with SCI. They performed a double-blinded, randomized controlled trial in 15 patients, and the results showed that the combination of morphine and clonidine demonstrated better pain relief than morphine or clonidine alone compared to placebo. Even though the results are statistically significant, the sample size remains small, and more studies need to be conducted on the effects of these medications on patients with SCI pain.

**Figure 2:**
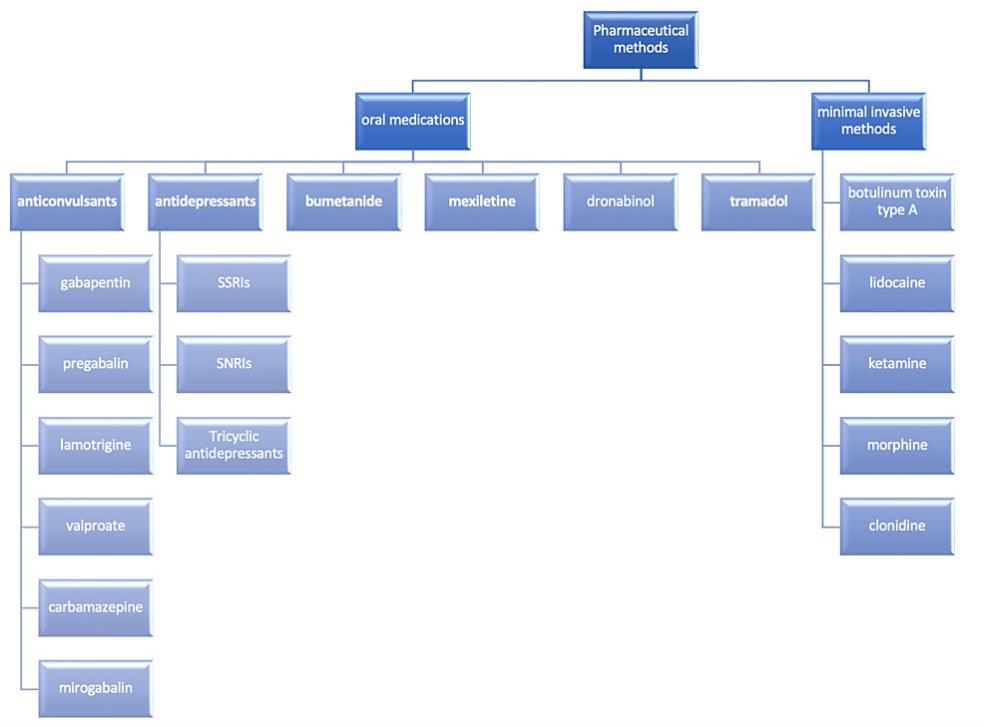
Pharmaceutical methods for the management of chronic pain in patients after SCI Abbreviations: SSRIs, selective serotonin reuptake inhibitors; SNRIs, serotonin and norepinephrine reuptake inhibitors; SCI, spinal cord injury

Figure 2 shows a synopsis of all the pharmaceutical methods used to manage chronic pain in patients after SCI.

Physical Therapy and Alternative Methods

From the 16 studies found to examine the effect of physical therapy interventions and alternative methods on neuropathic pain, four used low-frequency TENS. Özkul et al. [63] in a randomized cross-over trial of 24 participants analyzed the results after the application of visual illusion and TENS for two weeks each in both groups and found a more significant decrease in pain intensity after TENS application than visual illusion. The studies conducted by Celik et al. [64], Bi et al. [55], and Vitalii and Oleg [56] found that TENS had resulted in a significant reduction of neuropathic pain levels compared to placebo TENS with the difference that Vitalii and Oleg [56] used it in combination with gabapentin.

Arienti et al. [53] compared OMT, pregabalin, and OMT in combination with pregabalin in 47 patients, and the results showed that all the interventions reduced pain, and in the group in which OMT was used in addition to medication, the pain relief was greater. Auricular acupuncture was also researched by Estores et al. [62], and their results showed a significant pain reduction.

Hicks et al. [52] in a 34-participant trial suggested an exercise protocol including both aerobic and loads training aiming at the reduction of pain and depression and found a statistically significant difference between the exercise and non-exercise groups. On the same base, Mulroy et al. [51] compared home-based strengthening, optimal movements, stretching, and education on transfers, raises, and wheelchair propulsion to 1 hour of educational video in 80 SCI patients with painful shoulders. The results showed that the intervention was effective in reducing long-standing shoulder pain and that the patients had a better overall quality of life.

Contradictory results were found about the effect of mental imagery techniques on neuropathic pain. Kaur et al. [57] found noteworthy differences in pain intensity between mental imagery and especially laterality training and sham treatment. Also, Zanca et al. [49] used meditation in addition to mental imagery but showed that pain interference decreased more in the control group than the intervention group. A possible explanation could be that because the direct effects of mental imagery are expected to be on modification of the pain experience rather than pain interference itself, the effects on pain interference are likely to be indirect and influenced by other factors unrelated to the intervention. Additionally, Lovas et al. [59] found massage therapy as effective as guided imagery relaxation on pain relief. An interesting study by Jensen et al. [54] concluded that hypnosis was as effective as biofeedback relaxation in decreasing neuropathic pain, although the analgesia was maintained until the three-month follow-up only in the first group.

New techniques in rehabilitation have also emerged as Austin et al. [50] found that 3D head-mounted virtual reality was linked to greater levels of patients’ awareness as well as pain relief compared to 2D screen application. Furthermore, Burke et al. [58] researched a self-reported internet-delivered six-module CBT pain management program and demonstrated a substantial short-term effect on pain intensity, with pain benefits lasting three months.

An anti-inflammatory diet might affect the inflammation related to neuropathic pain. Allison et al. [60] showed that reducing inflammation as a treatment method for neuropathic pain in SCI is effective, with a possible mechanism involving a decrease in pro-inflammatory cytokines and prostaglandin E2. The effects of ultra-micronized palmitoylethanolamide as an additional treatment for neuropathic pain in people with SCI were studied by Andresen et al. [61]. However, they discovered no significant difference in pain intensity between ultra-micronized palmitoylethanolamide and placebo treatments, and ultra-micronized palmitoylethanolamide had no discernible effects on spasticity, sleep problems, anxiety, melancholy, or overall well-being.

Limitations

This systematic review included only studies written in English and Greek. One significant limitation is the limited number of studies and a small number of participants especially in minimally invasive methods and physical therapy and alternative treatments. That reduces the statistical power and increases the risk of bias. The small participant numbers also limit the ability to detect subtle effects and potential variations in treatment responses.

The heterogeneity in some study designs, especially in physical therapy and alternative interventions, and outcome metrics across the different trials make direct comparison and synthesis challenging. This variability makes it difficult to draw definitive conclusions and develop standardized treatment protocols. It is important to also note that some studies relied on self-report measures, which can be subject to biases and subjective interpretations by the participants. Objective measures and longer-term follow-up are necessary to strengthen the validity of the findings. Furthermore, incorporating diverse populations and utilizing more objective metrics would enhance the ability to generalize reliable data; therefore, physicians could use them in everyday practice.

## Conclusions

Several treatment options can reduce pain in patients after SCI. Now there is strong evidence that anticonvulsants and more specifically gabapentin have a beneficial role in managing chronic neuropathic pain in these patients and that gabapentin is considered as first-line treatment. Pregabalin seems to be an effective alternative as it also reduces patients' anxiety. On the other hand, serotonin and noradrenaline reuptake inhibitors seem ineffective in treating neuropathic pain in patients with SCI, and the results of tricyclic antidepressants (amitriptyline) were controversial.

rTMS and tDCS in some studies were shown to reduce pain intensity, although more studies with larger samples and with “higher” methodological quality should be conducted. All the minimally invasive methods significantly reduced pain intensity. Physical therapy and alternative methods especially TENS, OMT, and exercise therapy seem to be beneficial in managing chronic neuropathic pain after SCI. Finally, virtual reality is a very promising treatment for pain management and further studies for this method should be conducted.
